# Agreement between chest radiograph and computed tomography for the diagnosis of acute respiratory distress syndrome: A retrospective observational study

**DOI:** 10.1016/j.aicoj.2026.100172

**Published:** 2026-09-30

**Authors:** Jérémy Caldonazzo, Guillaume Herpe, Maïa Joos, François Arrivé, Chloé Villaret, Sylvain Le Pape, Vincent Menguy, Anne Veinstein, Jean-Pierre Frat, Arnaud W. Thille, Rémi Coudroy

**Affiliations:** a Service de Médecine Intensive Réanimation, CHU de Poitiers, Poitiers, France; b Service de Radiologie, CHU de Poitiers, Poitiers, France; c Laboratoire de Mathématiques Appliquées LMA, CNRS UMR 7348, Poitiers, France; d INSERM CIC 1402, IS-ALIVE research group, Université de Poitiers, Poitiers, France

**Keywords:** Acute respiratory distress syndrome, Diagnostic imaging, Computed tomography, Chest radiograph, Sensitivity, Specificity

## Abstract

**Background:**

The presence of bilateral lung infiltrates on chest imaging is a mandatory clinical criterion for the diagnosis of acute respiratory distress syndrome (ARDS). Although lung infiltrates are usually identified on chest radiograph in clinical practice, chest computed tomography (CT) outperforms chest radiograph for the diagnosis of lung infiltrates. We hypothesized that the performance of chest radiograph for the diagnosis of ARDS, as compared to chest CT as reference, is limited.

**Methods:**

We conducted a retrospective, single-center, observational study over a 7-year period, including patients who met all but the imaging criterion for ARDS (defined by the Berlin criteria) and who underwent a chest CT and a chest radiograph the same day. Chest CT was interpreted by two radiologists and considered as the gold standard diagnosis of ARDS whereas chest radiographs were interpreted by two intensivists. The primary outcome was the agreement between chest radiograph and chest CT for the diagnosis of ARDS in the overall population. Secondary outcomes included the performance of chest radiograph for the diagnosis of ARDS in the overall population and in predefined subgroups, especially based on patient characteristics, imaging features, timing between chest radiograph and CT, and inter-rater agreement. Agreement was assessed using Cohen’s kappa, and diagnostic performance using sensitivity, specificity, predictive values, and accuracy for the primary outcome and subgroup analyses.

**Results:**

Out of the 277 patients included during the study period, prevalence of ARDS on chest CT among hypoxemic intubated patients fulfilling all but the imaging criteria for ARDS was 81%. Overall, the agreement was fair to moderate and consistent across subgroups. Sensitivity of chest radiograph for the diagnosis of ARDS ranged from 74% to 80%, higher in patients with four quadrants involved on chest radiograph (100%), and lower in patients with focal ARDS on chest CT (54–67%).

**Conclusion:**

Chest radiograph has limited performance for the diagnosis of ARDS, and at least 20% of patients with ARDS on chest CT were not diagnosed using chest radiograph. Systematic chest CT in hypoxemic intubated patients may reduce the under-recognition of ARDS and contribute to effective implementation of protective ventilation strategies.

## Background

Acute respiratory distress syndrome (ARDS) is characterized by bilateral inflammatory lung edema. It accounts for around 25% of all patients invasively ventilated in intensive care units (ICUs) and is associated with high mortality rate, reaching 40% in the most severe cases [1]. The definition of ARDS has evolved over time from the initial description by Ashbaugh and colleagues in 1967 [2], to the lung injury score of Murray and colleagues in 1988 [3], followed by the American-European conference consensus guidelines in 1994 [4], and finally the Berlin definition in 2012 [5]. Of note, the recent new global definition of ARDS did not change the definition of ARDS in intubated patients, but rather extended it to patients not intubated and to resource-limited settings [6]. According to the Berlin definition, ARDS in intubated patients is defined as the acute worsening of respiratory symptoms occurring within seven days after a known clinical insult and responsible for hypoxemia with partial pressure of arterial oxygen (PaO_2_) to inspired fraction of oxygen (FiO_2_) equal to or below 300 mm Hg despite positive end-expiratory pressure equal to or above 5 cm H_2_O, unrelated to cardiac failure or fluid overload, and associated with bilateral lung infiltrates on chest imaging, *i.e.* on chest radiograph or chest computed tomography (CT) [5]. Although the Berlin definition for ARDS is pragmatic, It has been shown that ARDS is not recognized by physicians in approximately 40% of cases, which may lead to inappropriate ventilator settings and monitoring [1].

The radiological item is the most subjective criteria of the Berlin definition of ARDS and may help to explain the low recognition rate of ARDS [7]. First, interobserver agreement for the diagnosis of bilateral infiltrates on chest radiograph is slight to fair between intensivists [8], and even between ARDS experts [9,10]. Second, interobserver agreement for the diagnosis of ARDS between chest radiograph and chest CT, both of them interpreted by radiologists is only fair [11]. Moreover, chest radiographs in ICUs are rarely interpreted by radiologists, suggesting that interobserver agreement for the diagnosis of ARDS between chest radiograph interpreted by the intensivist and chest CT interpreted by the radiologist could be even poorer. Although chest CT could outperform chest radiograph in diagnosing lung infiltrates, a large-scale international study showed that only one quarter of patients with ARDS underwent a chest CT [12]. However, in patients with clinical suspected pneumonia admitted to an Emergency Department with no infiltrates on chest radiograph, about one-third of patients have infiltrates on chest CT [13].

We hypothesized that for the diagnosis of ARDS, compared to the chest CT interpreted by the radiologist, the reliability of the chest radiograph interpreted by the intensivist is poor.

## Methods

### Study design and participants

We conducted a single-center, retrospective, observational study including all patients admitted to the medical ICU at the University Hospital of Poitiers between January 1st, 2015, and December 31st, 2021 who had acute onset or worsening of hypoxemic respiratory failure within 1 week and who underwent a chest CT while intubated and whose PaO_2_/FiO_2_ was ≤300 mm Hg on the day of the chest CT. Patients who could not meet the criteria for ARDS according to the Berlin definition the day of the chest CT, *i.e.* who were ventilated with PEEP below 5 cm H_2_O, and those whose final diagnosis was cardiogenic pulmonary edema were excluded [5]. Additionally, we excluded patients who did not undergone a chest radiograph within the 24 h before or after the chest CT. The study was approved by the independent institutional review board of Poitiers and registered at http://www.clinicaltrials.gov (NCT07400445). In compliance with the national legislation regarding observational retrospective studies, and the study was declared to European General Data Protection Regulation (MR-004). An information letter was sent to all patients eligible in the study, and patients could be included on the condition that they did not object.

### Study setting

Chest radiographs were interpreted by intensivists whereas chest CT scans were interpreted by radiologists, reflecting pragmatic daily practice. Although both were blinded from the patients’ medical history, clinical and biological data, they were informed that all patients fulfilled the criteria for ARDS according to the Berlin definition, except the imaging one [5].

Chest CTs were performed according to international standards [14], with slice thickness between 0.6 mm and 1.25 mm, mean ± SD pitch of 1.25 ± 0.2, mean ± SD voltage of 120 ± 40 kVp, with automated milliamp modulation. Mean irradiation was 120 ± 30 mGy.cm.

For each patient, chest CTs were anonymized and interpreted by a senior radiologist with 10 years of experience and a resident radiologist with two years of experience. For each chest CT, lung fields were divided into four quadrants (upper right, upper left, lower right including middle and lower right lobes, and lower left). In line with the Berlin and the global definitions of ARDS, bilateral opacities on chest CT were considered only if they were not fully explained by effusions, atelectasis, or nodules/masses [5,6].

For each quadrant, the extent of ground-glass opacities and consolidations was analyzed semi-quantitatively (<25%, 25–50%, 50–75%, and >75%) by consensus between radiologists. ARDS on chest CT was defined by the extent of infiltrates (either ground-glass opacities or consolidations) ≥ 25% in one of the right quadrants and in one of the left quadrants [15]. Focal ARDS was defined as bilateral infiltrates with no infiltrates in the upper quadrants on chest CT. Diffuse ARDS on chest CT was defined as infiltrates ≥ 25% in the four quadrants on chest CT.

For each patient, the chest radiograph performed at the time closest to the chest CT was anonymized and interpreted by a fellow intensivist with five years of experience in the ICU (Rater #1), and a senior intensivist with 20 years of experience in the ICU (Rater #2). For each chest radiograph, lung fields were divided into four quadrants (upper right, upper left, lower right and lower left). For each quadrant, lung infiltrates were analyzed semi-quantitatively (0: no abnormality, 1: interstitial infiltrates, 2: non-confluent alveolar infiltrates, 3: confluent alveolar infiltrates), by each intensivist, one independently from the other [16,17]. ARDS on chest radiograph was defined by a score ≥ 1 in one of the right quadrants and in one of the left quadrants.

### Data collection

Demographic characteristics, comorbidities including underlying chronic cardiac disease, underlying respiratory disease, and immunosuppression [18] were collected.

Characteristics during ICU stay such as risk factors for ARDS [19], severity scores including Simplified Acute Physiology Score (SAPS) II [20] and Sequential Organ Failure Assessment score [21], time from ICU admission to intubation, treatments during ICU stay such as neuromuscular blocking agents, prone positioning, corticosteroids and ECMO, and outcomes such as duration of mechanical ventilation, duration of ICU stay and mortality at day 28 from ICU admission were collected.

Characteristics for the day of the chest CT including time from intubation to chest CT, time from chest radiograph to chest CT, PaO_2_/FiO_2_ the day of the chest CT, and the last ventilator settings before the chest CT (mode of ventilation, tidal volume, positive end-expiratory pressure, and respiratory system compliance for patients ventilated in volume-controlled mode) were collected.

### Objectives

The primary objective was to measure the level of agreement for the diagnosis of ARDS according to the Berlin definition in the overall population between chest radiographs interpreted by intensivists and chest CTs interpreted by radiologists.

Secondary objectives were to measure 1) the performance of chest radiograph for the diagnosis of ARDS, the level of agreement for the diagnosis of ARDS according to the Berlin definition between chest radiograph interpreted by intensivists and chest CT interpreted by radiologists in the following subgroups; 2) in patients with moderate-to-severe hypoxemia defined as PaO_2_/FiO_2_ ≤ 200 mm Hg the day of the chest CT; 3) in immunocompromised patients; 4) in obese patients (body-mass index ≥ 30 kg/m^2^); 5) in patients with four quadrants involved on chest radiograph; 6) in the subgroup of patients with ARDS on chest CT defined by the extent of infiltrates (either ground-glass opacities or consolidations) ≥ 50% in one of the right quadrants and in one of the left quadrants; 7) according to the time elapsed between the chest radiograph and the chest CT; 8) in patients with non-confluent or confluent alveolar infiltrates on chest radiograph (radiograph score ≥ 2); 9) in patients with pleural effusion on chest CT; and 10) in patients without SARS-COV-2 infection. We also assessed the performance of chest radiograph interpreted by the intensivists for the diagnosis of ARDS according to the Berlin definition 11) in patients with focal, 12) in patients with diffuse ARDS on chest CT, and 13) the agreement between intensivists for the diagnosis of ARDS on chest radiograph.

### Endpoints

The primary endpoint was the Cohen's kappa coefficient in the overall population, which quantifies agreement between two observers and ranges between 0 and 1 (0 to 0.20: slight agreement, 0.21 to 0.40: fair agreement, 0.41 to 0.60: moderate agreement, 0.61 to 0.80: substantial agreement, and 0.81 to 1: almost perfect agreement) [22].

Secondary endpoints were 1) sensitivity, specificity, positive and negative predictive value, and diagnostic accuracy of the chest radiograph for the diagnosis of ARDS, and the Cohen's kappa coefficient in the different subgroups of patients, *i.e.* 2) in patients with PaO_2_/FiO_2_ ≤ 200 mm Hg; 3) in immunocompromised patients; 4) in obese patients; 5) in patients with four quadrants involved on chest radiograph; 6) in ARDS patients with bilateral infiltrates extent ≥ 50% on chest CT; 7) in patients whom chest radiograph was performed within 6 h of the chest CT, between 6 and 12 h, and between 12 and 24 h; 8) in patients with alveolar infiltrates on chest radiograph; 9) in patients with pleural effusion on chest CT; and 10) in patients without SARS-COV-2 infection. Sensitivity of chest radiograph to diagnose ARDS was also assessed 11) in patients with focal; 12) in patients with or diffuse ARDS on chest CT; and 13) the Cohen’s kappa coefficient for the diagnosis of ARDS on chest radiograph between intensivists.

### Sample size calculation

Assuming prevalence of ARDS on chest CT among hypoxemic intubated patients of 70% [23], we hypothesized that the Cohen’s kappa coefficient for the diagnosis of ARDS between chest radiograph interpreted by intensivists and chest CTs interpreted by radiologists would be 0.20 (representing slight agreement), lower than the Cohen’s kappa coefficient of 0.36 for the diagnosis of ARDS between chest radiograph and chest CT, both of them interpreted by radiologists [11]. The inclusion of 326 patients would allow detection of slight agreement for the diagnosis of ARDS between chest radiograph interpreted by intensivists and chest CT interpreted by radiologists, with alpha risk of 5% and power of 80% [24].

### Statistical analysis

Continuous variables were represented as mean ± SD (standard deviation) or median [25th–75th percentile] depending on their distribution and compared using Student's *t*-test or the Mann-Whitney test. Categorical variables were represented as number (percentage) and compared using Chi-squared or Fisher's exact test. Cohen's kappa coefficient [95% confidence interval], sensitivity, specificity, positive predictive value, negative predictive value, and diagnostic accuracy for the diagnostic of ARDS on chest radiograph were calculated for each intensivist, considering ARDS on chest CT as reference in the overall population and in each subgroup. Sensitivity of the chest radiograph for the diagnosis of ARDS was calculated in patients with focal or diffuse ARDS on chest CT (for these patients, specificity could not be calculated as all patients had ARDS on chest CT).

Two-tailed p values < 0.05 were considered significant. Statistical analysis was performed using R software version 4.0.4 (http://www.R-project.org).

## Results

Over the 7-year study period, between January 1st, 2015, and December 31st, 2021, 336 intubated patients underwent a chest CT in our unit. After excluding 59 patients who did not meet ARDS criteria or had missing data (Fig. 1), 277 patients who met all of the ARDS criteria according to the Berlin definition (*i.e.* hypoxemia defined as PaO_2_/FiO_2_ ratio ≤ 300 mm Hg with PEEP of at least 5 cm H_2_O under invasive mechanical ventilation not explained by cardiogenic pulmonary edema and occurring within seven days after a known clinical insult), with the exception of the radiological criterion were analyzed. All in all, prevalence of ARDS in our cohort of hypoxemic intubated patients on chest CT was 81% (223 out of 277 patients).

**Fig. 1:**
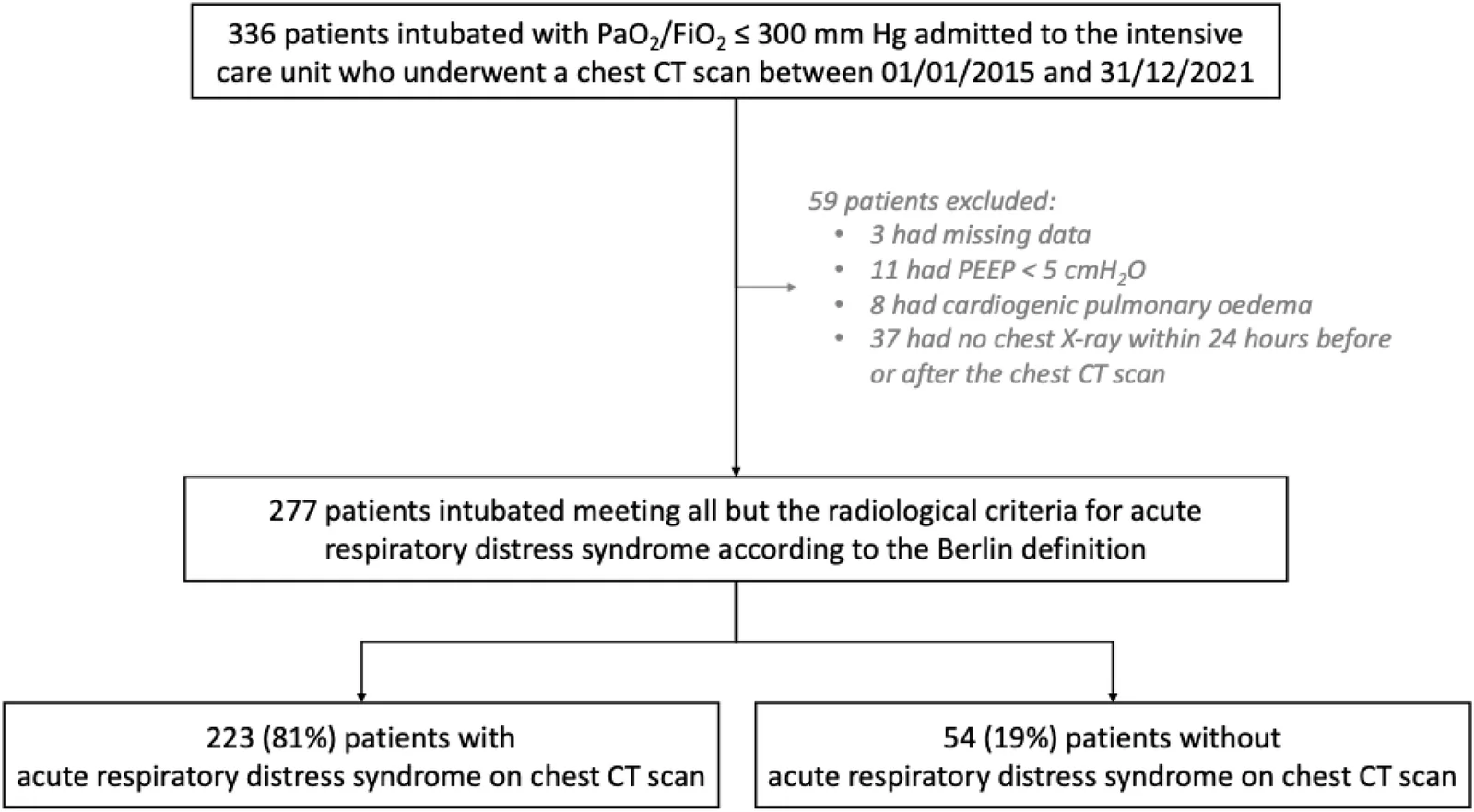
Flow Chart of patients intubated who underwent chest CT scan during the study period. CT: Computed Tomography; FiO2: fraction of inspired oxygen; PaO2: partial pressure of arterial oxygen; PEEP: Positive End Expiratory Pressure.

The patients included had a mean age of 62 ± 14 years and 72 (26%) were females. At ICU admission, mean SAPS II was 53 ± 21 points. The main clinical insult for ARDS was pneumonia in 205 patients (74%). During their ICU stay, 167 patients (60%) received neuromuscular blockers and 101 (36%) underwent prone positioning. Median durations of mechanical ventilation and ICU stay were 12 [6–22] and 15 [8–28] days, respectively. In-ICU mortality was 37% (103 patients). As compared to patients who had no ARDS on chest CT, patients with ARDS (bilateral lung infiltrates) were more likely to have SARS-CoV-2 infection (31 patients), were more hypoxemic and had lower respiratory system compliance (Table 1). The chest CT was performed earlier after intubation in patients with ARDS.

**Table 1:** Comparison of characteristics and outcomes between patients with and without ARDS diagnosed on chest CT.

	No ARDS	ARDS	p value
	N = 54	N = 223	
**Baseline characteristics**			
Age, years	67 [57−74]	63 [54−71]	0.0770
Sex, woman, n (%)	13 (24%)	59 (26%)	0.8529
Body-mass index, kg/m²	27 [23–31]	27 [23–32]	0.6039
Obesity, BMI ≥ 30 kg/m^2^, n (%)	19 (35%)	76 (34%)	1.0000
Comorbidities, n (%)			
Cardiac, n (%)	13 (24%)	53 (24%)	1.0000
Respiratory, n (%)	8 (15%)	33 (15%)	1.0000
Immunosuppression	15 (28%)	65 (29%)	0.9745
Hematological malignancy	7 (13%)	31 (14%)	1.0000
Leukopenia or lymphopenia	3 (6%)	16 (7%)	0.9026
**Characteristics at ICU admission**			
Simplified Acute Physiology Score (SAPS) II	54 [40−64]	50 [37−66]	0.2966
Sequential Organ Failure Assessment (SOFA) score	9 [6–12]	9 [6–13]	0.7867
Time from ICU admission to intubation, days	0 [0−0]	0 [0−1]	**0.0485**
Causes of ARDS, n (%)			
Pneumonia	35 (65%)	170 (76%)	0.1227
SARS-CoV-2	1 (2%)	30 (13%)	**0.0288**
Extra-pulmonary sepsis	27 (50%)	93 (42%)	0.3417
Non-cardiogenic shock	34 (63%)	131 (59%)	0.6802
Trauma	1 (2%)	4 (2%)	1.0000
Pulmonary contusion	0 (0%)	1 (0%)	1.0000
Drug intoxication	0 (0%)	1 (0%)	1.0000
Pulmonary vasculitis	0 (0%)	1 (0%)	1.0000
Pancreatitis	1 (2%)	9 (4%)	0.7148
Unknown	6 (11%)	13 (6%)	0.2812
**ICU stay**			
Neuromuscular blocking agent, n (%)	27 (50%)	140 (63%)	0.1171
Prone position, n (%)	17 (31%)	84 (38%)	0.4902
Steroids, n (%)	8 (15%)	55 (25%)	0.1712
Extracorporeal membrane oxygenation, n (%)	1 (2%)	15 (7%)	0.2925
**Characteristics on the day of the chest CT scan**			
Time between intubation and chest CT scan, days	5 [1–8]	1 [0−6]	**0.0096**
Time between CT scan and chest radiograph, hours	−2 [-8−4]	−3 [-6−6]	0.3947
Ventilator settings and respiratory mechanics			
Ventilation mode, n (%)			0.1533
Assist-Control Ventilation (ACV), n (%)	42 (78%)	193(86%)	
Pressure Support Ventilation (PSV), n (%)	12 (22%)	28 (13%)	
Airway Pressure Release Ventilation (APRV), n (%)	0 (0%)	2 (1%)	
Tidal volume, mL/kg predicted weight	6.6 ± 1.4	6.5 ± 1	0.4326
Positive end-expiratory pressure, cmH_2_O	8 ± 3	9 ± 3	0.3255
Respiratory system compliance (in ACV patients), mL/cmH_2_O	42 ± 21	33 ± 17	**0.0085**
PaO_2_/FiO_2_, mm Hg	184 ± 56	158 ± 60	**0.0046**
Severity of hypoxemia on day of chest CT, n (%)			**0.0060**
Mild (PaO_2_/FiO_2_ > 200 and ≤ 300 mm Hg)	23 (43%)	55 (24%)	
Moderate (PaO_2_/FiO_2_ > 100 and ≤ 200 mm Hg)	28 (52%)	124 (56%)	
Severe (PaO_2_/FiO_2_ ≤ 100 mm Hg)	3 (5%)	44 (20%)	
**Outcomes**			
Length of ICU stay, days	14 [8–28]	16 [8–28]	0.7239
Length of mechanical ventilation, days	12 [7–24]	12 [6–22]	0.8828
Mortality at day 28, n (%)	17 (31%)	86 (39%)	0.4182

ARDS: Acute Respiratory Distress Syndrome; ICU: Intensive Care Unit; CT: Computed Tomography scan.

Continuous variables are represented as mean ± standard deviation or median [25th–75th percentile] depending on their distribution. Categorical variables are represented in number (percentage).

Among the 223 patients with ARDS on chest CT, bilateral infiltrates were focal in 45 cases (20%), and diffuse (4 quadrants) in 134 cases (60%). Ground glass opacities on chest CT were almost evenly distributed in all four lung quadrants, whereas consolidations were more frequently distributed in the lower than in the upper quadrants (Fig. 2).

**Fig. 2:**
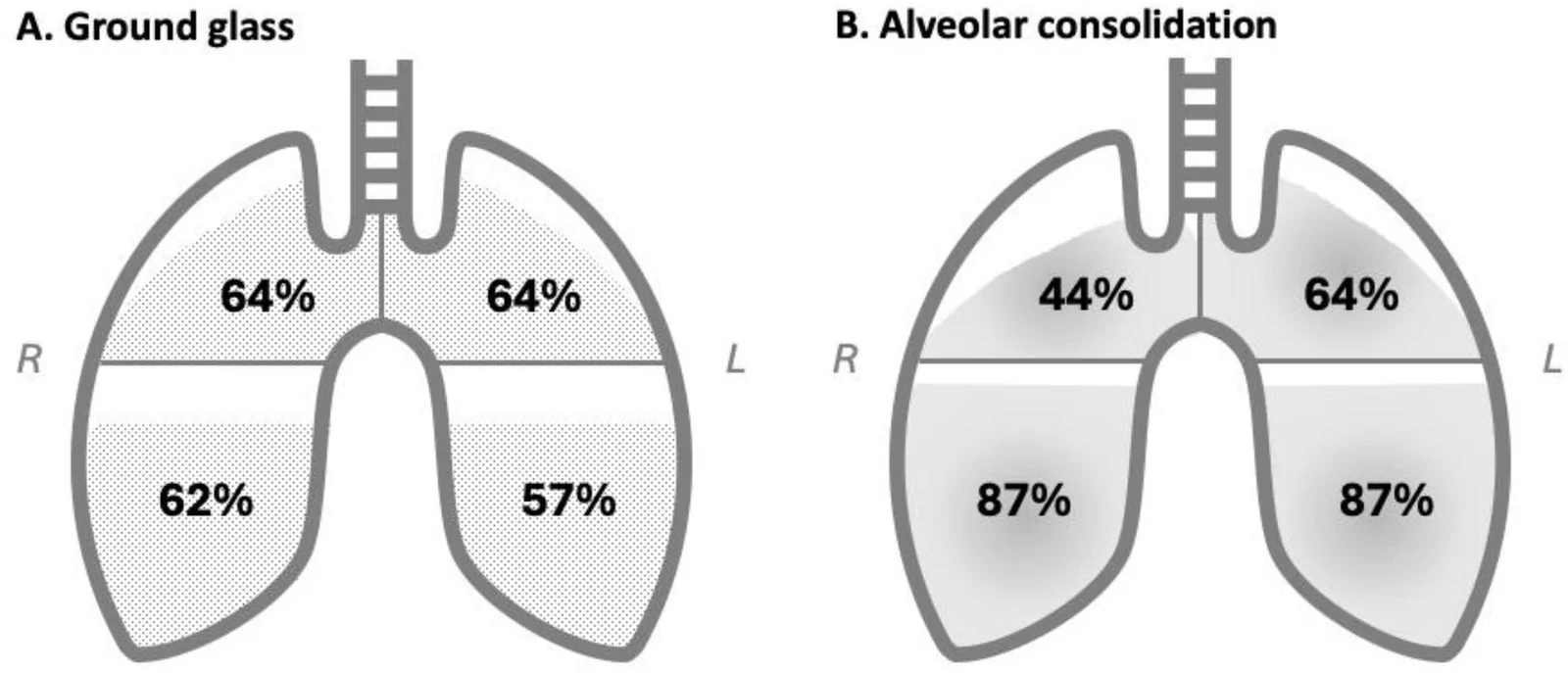
Distribution of lung infiltrates by quadrants among the 277 patients with ARDS on chest CT. Panel A: ground glass opacities, Panel B: consolidations. R: right; L: left.

### Agreement for the diagnosis of ARDS in the overall population (primary endpoint)

Among the 223 patients with ARDS on chest CT, raters #1 and 2 identified 203 (73%) and 188 (68%) patients with ARDS based on chest radiograph interpretation, respectively.

In the overall population, the agreement for the diagnosis of ARDS between the chest radiograph interpreted by intensivists and the chest CT interpreted by radiologists was fair to moderate with Cohen’s kappa of 0.29 (95% confidence interval 0.17−0.42) for Rater #1 and 0.44 (95% confidence interval 0.32−0.55) for Rater #2 (Table 2).

**Table 2:** Agreement between intensivist-interpreted chest radiographs and radiologist-interpreted chest CT for ARDS diagnosis.

	Cohen's Kappa coefficient	Sensitivity (%)	Specificity (%)	PPV (%)	NPV (%)	Diagnostic accuracy (%)
**Overall population (n = 277)**						
Rater #1	0.29 (0.17–0.42)	80 (74–85)	54 (40–67)	88 (82–92)	39 (28–51)	75 (69–80)
Rater #2	0.44 (0.32–0.55)	78 (72–84)	76 (62–87)	93 (88–96)	46 (35–57)	78 (73–83)
**Subgroup with PaO_2_/FiO_2_ ≤ 200 mmHg (n = 199)**						
Rater #1	0.17 (0.02–0.32)	79 (72–85)	42 (25–61)	88 (82–93)	27 (15–41)	73 (66–79)
Rater #2	0.39 (0.25–0.53)	80 (74–86)	71 (52–86)	94 (88–97)	40 (27–54)	79 (73–84)
**Subgroup with immunocompromised patients (n = 80)**						
Rater #1	0.26 (0.04–0.48)	74 (61–84)	60 (32–84)	89 (77–96)	35 (17–56)	71 (60–81)
Rater #2	0.40 (0.19–0.60)	74 (61–84)	80 (52–96)	94 (84–99)	41 (24–61)	75 (64–84)
**Subgroup with obese patients (n = 95)**						
Rater #1	0.30 (0.09–0.51)	78 (67–86)	58 (33–80)	88 (78–95)	39 (22–68)	74 (64–82)
Rater #2	0.43 (0.25–0.61)	74 (62–83)	84 (60–94)	95 (86–99)	44 (28–62)	76 (66–84)
**Subgroup of patients with ≥ 50% infiltrates extent on chest CT (n = 131)**						
Rater #1	0.25 (0.16–0.35)	87 (80–92)	39 (31–47)	56 (49–63)	77 (66–86)	62 (56–67)
Rater #2	0.37 (0.27–0.47)	88 (81–93)	50 (42–58)	61 (54–68)	82 (72 – 89	68 (62–73)
**Subgroup of patients with pleural effusion on chest CT (n = 213)**						
Rater #1	0.31 (0.17–0.46)	81 (75–87)	55 (38–71)	89 (83–94)	39 (26–53)	77 (70–82)
Rater #2	0.39 (0.25–0.53)	80 (73–86)	68 (51–82)	92 (87–96)	43 (30–56)	78 (72–83)
**Subgroup of patients without SARS-CoV-2 infection (n = 246)**						
Rater #1	0.28 (0.15–0.41)	77 (71–83)	55 (40–68)	86 (80–91)	40 (28–52)	72 (66–78)
Rater #2	0.43 (0.31–0.55)	77 (70–82)	75 (62–86)	92 (87–96)	47 (36–58)	76 (71–82)
**Subgroup with alveolar infiltrates on chest radiograph (n = 79 patients for Rater #1 and 102 for Rater #2)**						
Rater #1	0.15 (0.10–0.21)	35 (28–41)	96 (87–100)	97 (91–100)	26 (20–33)	47 (41–53)
Rater #2	0.17 (0.10–0.24)	43 (36–50)	89 (77–96)	94 (88–98)	27 (21–35)	52 (46–58)
**Subgroup with 4 quadrants involved on chest radiograph**$**(135 patients for Rater #1 and 78 for Rater #2)**						
Rater #1		100 (95–100)				
Rater #2		100 (97–100)				
Subgroup with focal ARDS on chest CT*, $**(n = 45)**						
Rater #1		67 (53–79)				
Rater #2		54 (40–67)				
Subgroup with diffuse ARDS on chest CT**, $**(n = 134)**						
Rater #1		93 (87–96)				
Rater #2		90 (84–95)				

ARDS: Acute Respiratory Distress Syndrome; CT: Computed Tomography scan; PPV: positive predictive value; NPV: negative predictive value.

True positives (TP) are patients for whom both the intensivist and the radiologist found bilateral infiltrates on chest radiograph and on chest CT.

True negatives (TN) are patients for whom both the intensivist and the radiologist found no bilateral lung infiltrates on both chest radiograph and chest CT.

False positives (FP) are patients for whom the intensivist found bilateral infiltrates on chest radiograph, while the radiologist found no bilateral infiltrates on chest CT.

False negatives (FN) are patients for whom the intensivist found no bilateral infiltrates on chest radiograph, while the radiologist found bilateral infiltrates on chest CT.

Diagnostic accuracy is calculated as (TP + TN)/(TP + TN + FP + FN).

$ Cohen’s kappa, and specificity could not be calculated given that all patients had ARDS on chest CT. Therefore, there was only TP and FN, but no TN or FP.

* Focal ARDS was defined as bilateral infiltrates in lower quadrants without infiltrates in the upper quadrants on chest CT.

** Diffuse ARDS was defined as bilateral infiltrates involving the four quadrants (either ground-glass opacities or consolidations on chest CT.

Sensitivity of chest radiograph for the diagnosis of ARDS was around 80%, meaning that 20% of ARDS diagnoses were missed based on chest radiograph interpretation. Specificities of chest radiograph for the diagnosis of ARDS were 54 and 76%, meaning that 46 and 24% of ARDS were overdiagnosed based on chest radiograph interpretation, respectively.

### Subgroup analyses

Results were consistent in the subgroup analyses including patients with PaO_2_/FiO_2_ ≤ 200 mm Hg the day of the chest CT, immunocompromised patients, obese patients, patients without SARS-CoV-2 infection, and patients with pleural effusion on chest CT (Table 2). In the subgroup of patients with four quadrants involved on chest radiograph, sensitivity of chest radiograph for the diagnosis of ARDS was 100%, meaning that all patients had ARDS on chest CT. Cohen’s kappa were consistent according to the time elapsed between chest radiograph and chest CT (Supplemental Table 1). When only bilateral infiltrates ≥ 50% were considered for the diagnosis of ARDS on chest CT, the prevalence of ARDS decreased to 47% (131 of 277 patients), leading to increased sensitivity (87% and 88%) but decreased specificity (39% and 50%). When only alveolar infiltrates were considered for the diagnosis of ARDS on chest radiograph, agreement (Cohen’s kappa 0.15 and 0.17, respectively) and sensitivity (35% and 43%, respectively) were low for both raters, whereas specificity was high (96% and 89%, respectively).

Among the 45 patients with focal ARDS on chest CT, sensitivity of chest radiograph for the diagnosis of ARDS was between 54 and 67%, lower than in the overall population, meaning that 33 and 46% of ARDS diagnoses were missed based on chest radiograph interpretation. Among the 134 patients with diffuse ARDS on chest CT, sensitivity of chest radiograph for the diagnosis of ARDS was around 90%, meaning that only 10% of ARDS diagnosis were missed based on chest radiograph interpretation (Table 2). The inter-rater agreement for the diagnosis of ARDS on chest radiograph between the two intensivists was moderate with Cohen’s kappa of 0.42 (95% confidence interval 0.30−0.54).

## Discussion

In our cohort of hypoxemic patients invasively ventilated who underwent a chest CT, prevalence of ARDS was 81%. The agreement between chest radiograph interpreted by intensivists and chest CT interpreted by radiologists for the diagnosis of ARDS was only fair to moderate, with acceptable sensitivity (78–80%), but specificity was moderate (54–76%). In patients with four quadrants involved on chest radiograph, sensitivity was perfect. In patients with focal ARDS on chest CT, up to 46% of ARDS were missed based on chest radiograph interpretation, whereas in patients with ARDS and four quadrants involved on chest CT, approximately 10% of ARDS were missed based on chest radiograph interpretation.

The prevalence of ARDS on chest CT in our cohort of hypoxemic intubated patients was high (81%), suggesting that patients were highly selected. This may have influenced the predictive values of chest radiograph for the diagnosis of ARDS. However, the prevalence of ARDS in hypoxemic intubated patients we report is in keeping with the 74% reported in a previous study including 90 patients with chest CT [11], suggesting that the prevalence of ARDS among hypoxemic intubated patients is actually high. Likewise, focal ARDS on chest CT accounted for 20% of ARDS in our cohort, close to the 27% reported in a prospective multicenter study including 119 consecutive ARDS who underwent chest CT [25]. In-ICU mortality rates of patients with and without ARDS were 39% and 31%, respectively, in line with the 35% and 29% reported in a large-scale international prospective cohort study including 3165 patients [23]. These findings reinforce the external validity of our results.

Overall agreement for the diagnosis of ARDS between the chest radiograph interpreted by intensivists and the chest CT interpreted by radiologists was fair to moderate. A similarly poor performance of the chest radiograph was reported in a study including 90 ICU patients in which chest radiograph and chest CT were both interpreted by radiologists [11]. As radiologists outperformed intensivists in the assessment of the Lung Injury Score on chest radiograph [8], lower agreement could therefore be expected in our study [11]. However, in a large-scale study including 3423 patients admitted to an Emergency Department, agreement between chest radiograph and chest CT, both interpreted by radiologists, for the diagnostic of lung infiltrates was only fair [26]. Likewise, we report sensitivities of the chest radiograph interpreted by intensivists for the diagnosis of ARDS ranging from 74% to 80% in the overall population and in subgroup analyses, higher than the sensitivities reported for the diagnosis of consolidations (from 38 to 60%) or of interstitial syndrome (from 46 to 68%) in studies in which chest radiograph and chest CT were interpreted by radiologists [27,28]. As chest radiographs performed in ICUs are often interpreted by intensivists [29], we believe that our study design in which chest radiographs were interpreted by intensivists aligns with clinical practice.

Whereas the distribution of ground glass opacities was even in the four lung quadrants, consolidations were more prevalent in the lower lung quadrants. Interestingly, in a study including 45 ICU patients, sensitivities for the diagnosis of lung consolidations and collapse on chest radiograph were poorer in the lower than in the upper lung quadrants [30]. There is a need for improving the diagnostic performance of chest radiograph, which remains challenging. The effect of education is debated [9,31,32]. Implementation of a semi-quantitative chest radiograph analysis such as the RALE score could be interesting [33], but it is poorly correlated with the chest CT severity score [34]. While combining independent assessment by multiple physicians improves the diagnostic performance of chest radiograph, it may be difficult in daily clinical practice [7]. The use of artificial intelligence is promising but not yet available in our units [35].

### Clinical implications

The main finding is that chest radiograph may not be sufficient for the diagnosis of ARDS. This is particularly true in patients with focal ARDS on chest CT in whom the sensitivity of chest radiograph decreased to only 54%, meaning that nearly half of patients were not diagnosed as ARDS. Furthermore, restricting the radiographic criterion to alveolar opacities on chest radiograph increased specificity from 54–76% to 89–96%, but reduced sensitivity from 78–80% to 35–43%, resulting in a substantial increase in false-negative diagnoses, thus making frank alveolar infiltrates on chest radiograph unsuitable as a screening criterion for the diagnosis of ARDS. Similarly, applying a stricter chest CT criterion (bilateral infiltrates involving ≥ 50% of quadrants) dramatically decreased the prevalence of ARDS, increased sensitivity from 78–80% to 87–88% but reduced specificity from 54–76% to 39–50%, resulting in a substantial increase in false-positive diagnoses. Except for patients with four lung quadrants involved on chest radiograph in whom the sensitivity of chest radiograph was 100%, our findings suggest that so as to look for bilateral infiltrates, a chest CT should be performed in all patients who remain hypoxemic under mechanical ventilation. Another option could be to assess the imaging criteria for ARDS using lung ultrasound which outperforms chest radiograph [36], and is henceforth included in the global definition of ARDS [6].

### Limitations

Some limitations must be acknowledged. First, we used chest CT as gold standard for the diagnosis of ARDS, whereas histological lesions of diffuse alveolar damage were initially the hallmark of ARDS [2]. However, less than half of patients meeting clinical criteria for ARDS and bilateral infiltrates on chest radiograph had diffuse alveolar damage at autopsy [37]. Moreover, a previous study reported that the sensitivity of chest radiograph for diagnosing diffuse alveolar damage on lung autopsy was 68% [38], close to the sensitivity of chest radiograph for diagnosing ARDS on chest CT we report herein. Second, the diagnosis of ARDS was established by radiologists based on chest CT, whereas chest radiographs were interpreted by intensivists. Consequently, the observed agreement reflects the combined effects of differences between imaging modalities and inter-rater variability, rendering it difficult to disentangle the respective contribution of these two sources of disagreement. However, agreement between the two intensivists assessing the same chest radiograph was of similar magnitude to that observed between each intensivist and the radiologists interpreting different imaging modalities. This finding suggests that the discordance observed between chest radiograph and CT may partly reflect interobserver variability, rather than being attributable solely to differences between imaging modalities. Importantly, the diagnostic accuracy of chest radiograph interpreted by intensivists for the diagnosis of ARDS (75% and 78%, respectively) was comparable to that reported in a previous study in which both chest radiographs and chest CT scans were interpreted by radiologists (72%) [11]. Likewise, inter-rater agreement for the diagnosis of ARDS on chest radiograph was moderate (Cohen’s kappa 0.42), slightly better than the fair agreement (Cohen’s kappa 0.30) reported for the analysis of chest radiographs [30]. Third, we did not test the inter-radiologist agreement for the diagnosis of ARDS on chest CT, which may question the reliability of our reference standard. However, the inter-rater agreement for the semi-quantitative assessment of lung infiltrates on chest CT by radiologists is high, suggesting our reference standard for the diagnosis of ARDS on chest CT is robust [39]. Fourth, our definition of focal ARDS slightly differed from that used in the LIVE trial [15]. In the LIVE trial, focal ARDS was defined as consolidations in the lower and back lung regions, whereas we defined focal ARDS as all types of infiltrates (including ground glass opacities) in the lower lung quadrants, which may have resulted in different diagnosis performance of chest radiograph. However, we reported that consolidations were mostly in the lower lung quadrants. Moreover, bilateral opacities required for the definition of ARDS are not restricted to consolidations, and may include ground-glass opacities [5,6]. Fifth, tidal volumes and PEEP influence the density of lung infiltrates [40,41], and we cannot rule out the possibility that different ventilator settings could have led to different results. However, the tidal volume and PEEP we found are in line with those reported in the largest multicenter cohort study on ARDS [1]. Sixth, the planned sample size was not reached (277 *vs*. 326 patients), resulting in a statistical power of 73%, lower than the 80% planned. Sixth, this study was retrospective with inherent selection bias. Seventh, raters were informed that patients met all the criteria for ARDS with the exception of the imaging one. Whether knowing the medical history, the clinical and biological data would have influenced their interpretation of chest radiograph and CT remains to be tested. Finally, we chose to include only patients susceptible to meet the criteria for intubated ARDS. Whether our findings are similar in non-intubated ARDS should be tested.

## Conclusion

Agreement between chest radiograph interpreted by intensivists and chest CT interpreted by radiologists for the diagnosis of ARDS was fair to moderate. In fact, approximately 20% of patients with ARDS were overlooked when only chest radiographs were analyzed by intensivists. This rate exceeded 30–40% in patients with focal ARDS. Consequently, a chest CT may help reducing the under-recognition of ARDS in mechanically ventilated patients with unexplained hypoxemia, with the final aim of implementing appropriate protective ventilation strategies.

## Authors' contributions

JC and RC analysed the data, performed the statistical analyses, and were majors’ contributors in writing the manuscript. GH and CV analysed the chest CT scans. MJ and VM collected the patients’ clinical data. FA, AV, and JPF interpreted the chest radiographs and reviewed the manuscript. SLP contributed to the statistical analyses. AWT analysed, reviewed and revised the manuscript. All authors read and approved the final manuscript.

## Ethics approval and consent to participate

The study was approved by the independent institutional review board of Poitiers. In compliance with the national legislation regarding observational retrospective studies, and the study was declared to European General Data Protection Regulation (MR-004). Clinical trial number was NCT07400445, and the study was registered on 6 February 2026.

## Consent for publication

An information letter was sent to all patients eligible in the study, and patients could be included on the condition that they did not object.

## Authors' information (optional)

Not applicable.

## Funding

Not applicable.

## Data availability

All data generated or analysed during this study are included in this published article.

## Declaration of competing interest

The authors declare that they have no competing interests.
