# Supplementary material for: Agreement between chest radiograph and computed tomography for the diagnosis of acute respiratory distress syndrome: A retrospective observational study
Source: Ann Intensive Care. 2026 Sep 30;16:100172. doi: 10.1016/j.aicoj.2026.100172 (PMC13675848; doi:10.1016/j.aicoj.2026.100172)
Supplement: Supplementary file 1 [file mmc1.docx]

**Supplemental Table 1. Agreement for the diagnosis of ARDS according to the time elapsed between chest radiograph and chest CT in the overall population.**

|  | **Cohen's Kappa coefficient** | **Sensitivity (%)** | **Specificity (%)** | **PPV (%)** | **NPV (%)** | **Diagnostic accuracy (%)** |
| --- | --- | --- | --- | --- | --- | --- |
| **-6 to +6h (n = 127)** | | | | | |  |
| Rater #1 | 0.31 (0.13 – 0.49) | 79 (69 – 86) | 58 (37 – 78) | 89 (81 – 95) | 39 (23– 57) | 75 (66 – 82) |
| Rater #2 | 0.41 (0.25 – 0.56) | 73 (63 – 81) | 83 (63 – 95) | 95 (88 – 99) | 42 (28 – 57) | 75 (66 – 82) |
| **-12 to -6h and +6 to +12h (n = 75)** | | | | | |  |
| Rater #1 | 0.26 (0.01 – 0.51) | 84 (73 – 93) | 41 (18 – 67) | 83 (71 – 92) | 44 (20 – 70) | 75 (63 – 84) |
| Rater #2 | 0.56 (0.34 – 0.78) | 88 (77 – 95) | 71 (44 – 90) | 91 (80 – 97) | 63 (38 – 84) | 84 (74 – 91) |
| **-24 to -12h and +12h to +24h (n = 75)** | | | | | |  |
| Rater #1 | 0.31 (0.07 – 0.54) | 77 (65 – 87) | 62 (32 – 86) | 91 (79 – 97) | 36 (17 – 59) | 75 (63 – 84) |
| Rater #2 | 0.38 (0.15 – 0.61) | 79 (67 – 88) | 69 (39 – 91) | 92 (82 – 98) | 41 (21 – 64) | 77 (66 – 86) |
